# Thermomechanical Properties of Carbon Nanocomposites PEGDA Photopolymers

**DOI:** 10.3390/molecules27206996

**Published:** 2022-10-18

**Authors:** Panagiotis Loginos, Anastasios Patsidis, Katerina Vrettos, George Sotiriadis, Georgios C. Psarras, Vassilis Kostopoulos, Vasilios Georgakilas

**Affiliations:** 1 Department of Material Science, University of Patras, University Campus, GR-26504 Rion, Greece; 2 Department of Mechanical Engineering and Aeronautics, University of Patras, University Campus, GR-26504 Achaia, Greece

**Keywords:** thermomechanical properties, PEGDA, graphene hybrid, carbon nanotubes, nanocomposites

## Abstract

In this work, UV-curable resin poly (ethylene glycol) diacrylate (PEGDA) was reinforced with three different types of nanofillers: pristine graphene (G), multiwalled carbon nanotubes (MWNTs), and a hybrid of MWNTs and graphene 70/30 in mass ratio (Hyb). PEGDA was mixed homogenously with the nanofiller oligomer by shear mixing and then photopolymerized, affording thin, stable films. The thermomechanical properties of the afforded nanocomposites indicated the superior reinforcing ability of pristine graphene compared with MWNTs and an intermediate behavior of the hybrid.

## 1. Introduction

Photopolymerization is a widely applied technique in coatings, inks, adhesives, biomaterials and, more importantly, has contributed remarkably in the rapid development of 3D printing imaging technology [1,2,3,4,5,6]. In the last few decades, the biomaterials and additive manufacturing (AM) industries have developed products with desirable properties and complex geometries. The main advantages of photopolymerization are a fast-adjustable curing process, and its eco-friendly nature, since it can be completed in the absence of solvents [6]. AM is a rapidly developing field and using conventional polymers provides excellent prototypes. However, often polymers need to be reinforced with proper fillers, to print items with advanced properties [7]. Fiber-reinforced photopolymers have been shown to exhibit the shape memory effect [7]. Photopolymers are often reinforced with ceramics [8], glass fiber [9], and inorganic fillers [10] mainly to improve their mechanical properties.

Reinforcement of photopolymers used by the two main 3D printing methods—laser based stereolithography (SLA) and digital light processing (DLP)—has not yet been studied and developed extensively. The main reason for this is that photopolymerization is a sequential process which starts from the surface, and therefore any filler embedded in a photopolymer tends to degrade the material, impeding double C=C bonds to be converted [11,12]. The addition of nanoparticles, including carbon-based nanofillers, such as carbon black, graphene nanosheets, or carbon nanotubes in a resin, can significantly improve the electrical, thermal, or mechanical properties of the resultant polymeric nanocomposites, providing great potential for 3D-printed nanocomposites for application in electromagnetic shielding, stretchable sensors, biosensors, and energy storage devices [13,14,15,16,17,18].

Although several graphene nanocomposites have been described so far, one of the key aspects to ensure efficient reinforcement from these nanofillers is the achievement of a high level of dispersion without obvious agglomeration. Because of their high specific surface area and surface chemical features, graphene sheets tend to be arranged in agglomerates or stacks due to van der Waals interactions [12,19]. It has been demonstrated that the formation of a nanoparticles network within the polymer matrix is a dynamic process that depends strongly on the mixing methods and viscosity of the polymer. The properties of the nanocomposite are a subsequent effect of this network’s formation [20,21,22].

In a previous work, we have used graphene (G), multiwalled carbon nanotubes (MWNTs), and a hybrid of MWNTs and graphene 70/30 in mass ratio (Hyb) as nanofillers of the UV-curable resin, poly (ethylene glycol) diacrylate (PEGDA), to study the influence of reinforcement on the electrical properties of the nanocomposites [23]. In this article, we examine the thermomechanical properties of the same nanocomposites, denoted as PEGDA/G, PEGDA/M, PEGDA/Hyb, respectively. The electrical properties of the polymer nanocomposites are an important parameter, for their exploitation in several applications. However, thermomechanical properties are of high importance as regards the stability, the working conditions, and the efficiency of the potential devices. Therefore, the aim of this work is to develop a novel series of photopolymer nanocomposites, combining PEGDA with the most common carbon nanomaterials such as 2D graphene, 1D carbon nanotube, and their hybrid, and to examine their role as regards thermomechanical properties.

## 2. Results and Discussion

The mass percentage of the reinforced nanocomposites was selected to be between 1.5 and 8.5% (*w*/*w*), taking into consideration their conductive behavior, as was recently presented [23]. The percentage of 1.5% *w*/*w* was selected as the lower percentage of the fillers, since this was the lowest ratio that surface electrical conductivity was recorded as in the composites. The higher percentage selected was 8.5% *w*/*w*. At this percentage, all samples were conductive and created stand-alone solid structures. At a higher percentage of the filler, very fragile and unstable structures were formed [23]. An intermediate percentage was included for better understanding of the behavior of the reinforced samples. Regarding the amount of PI, 2% *w*/*w* was selected because this has shown the best combination of properties [12]. The components—PEGDA oligomer, PI and carbon nanofiller—were mixed, forming a viscous liquid that was homogenized as much as possible using a handmade shear mixing technique. Then, the samples were placed between two glass plates and irradiated with a UV lamp (16 Watt) for 15 min. In the next table, the as-prepared composite samples are presented (see Table 1). The neat PEGDA with 2% *w*/*w* PI was also prepared as a reference sample.

**Figure 1:**
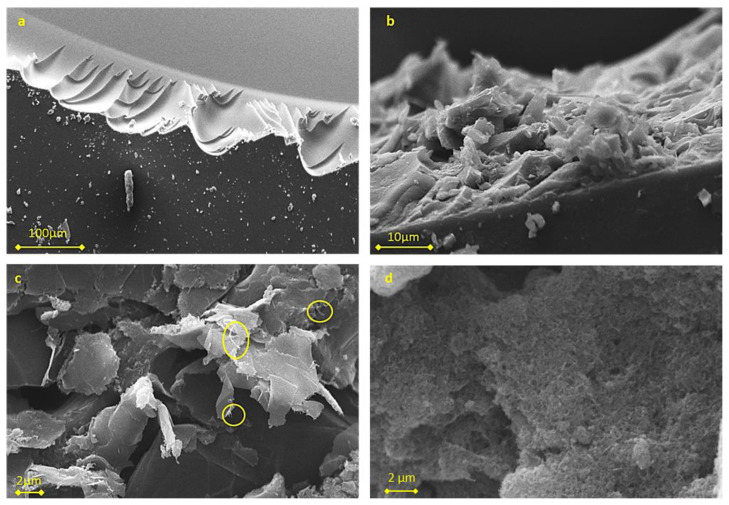
SEM images from the internal space of (**a**) Neat PEGDA polymer and PEGDA reinforced with (**b**) Graphene (**c**) Hybrid and (**d**) MWNTs. In image (**c**) some MWNTs that are visible in the surface have been marked.

The morphology of the composite samples with 8.5% (*w*/*w*) nanofiller were observed in SEM microscopy images, depicted in Figure 1. In the cross-section of the neat polymer film (Figure 1a), a smooth surface is observed in contrast with the rough internal texture observed in the reinforced samples (Figure 1b–d), that indicates the uniform dispersion of the nanofillers by the successful high-shear mixing of the components before photo curing.

**Figure 2:**
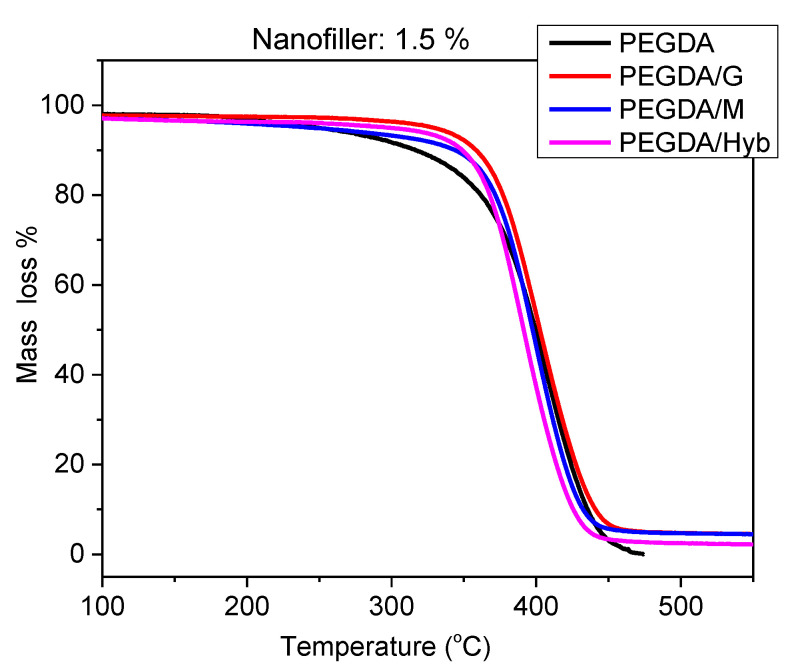
TGA for Neat PEGDA and PEGDA/G, PEGDA/M and PEGDA/Hyb composites, at 1.5% *w*/*w*.

TGA diagrams (Figure 2) were analyzed to examine the thermal stability of the reinforced samples. Due to the low percentage of the nanofillers, the thermal behavior of the reinforced samples is much similar to that of the neat photopolymer [24]. Neat PEGDA starts to be degraded at 300 °C. Regarding the samples with 1.5% *w*/*w* nanofiller, the polymer was protected from degradation below 340 °C, especially the samples with graphene (PEGDA/G), probably due to the extended interaction of the polymer with the surface of graphene. However, at higher temperature, the degradation of the reinforced samples is comparable with that of the neat polymer [24,25]. Similar behavior was observed within the samples of the rest of the percentages.

In Table 2, the glass transitions (T_g_) of the samples were presented, as calculated by DSC measurements. The T_g_ of the reinforced with MWNTs samples PEGDA/M decreased. This is attributed to the increased mobility of the polymer chains, due to weaker interactions that are caused by the presence of carbon nanotubes between the chains [24]. On the other hand, T_g_ of PEGDA/G and PEGDA/Hyb samples increased slightly in all percentages. Here, the polymer chains interact with the large surfaces of the 2D layered nanofiller, such as graphene with van der Waals forces, causing reduced mobility of the polymer network. This hypothesis was further confirmed by the fact that T_g_ decreased more intensely in reinforcement with pure graphene, PEGDA/G, and especially for the sample with the highest graphene percentage, instead of that of the hybrid PEGDA/Hyb [24].

**Figure 3:**
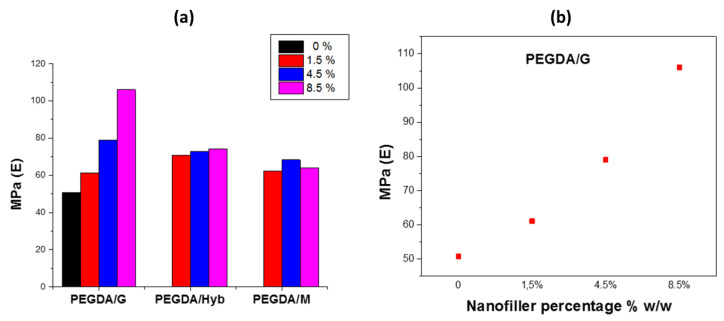
(**a**) Young’s modulus for different mass percentage of all materials. (**b**) Young’s modulus of PEGDA/G composites at different percentages.

Figure 3 shows the results of the tensile study of the reinforced samples in DMA. From the tensile tests, an improvement of the Young’s modulus (E) is observed for all the reinforced specimens. More specifically, PEGDA/G samples showed the most significant increase of the Young’s modulus, which increased, furthermore, almost linearly with the graphene percentage. The Young’s modulus for the sample of 8.5% *w*/*w* of graphene showed the maximum increase, up to 108%, compared with the neat PEGDA.

The reinforced with MWNTs samples PEGDA/M showed minimum improvement as regards the elastic modulus, with a mean increase of 28% for the three samples compared with the neat PEGDA. The PEGDA/Hyb specimens showed an intermediate behavior with a mean increase about 43.3%. Finally, the increase of the elastic modulus in the PEGDA/M samples seems to be independent from the percentage of the nanofiller in contrast with the PEGDA/G samples. This indicates a different behavior of MWNTs compared with graphene, regarding the polymer reinforcement.

**Figure 4:**
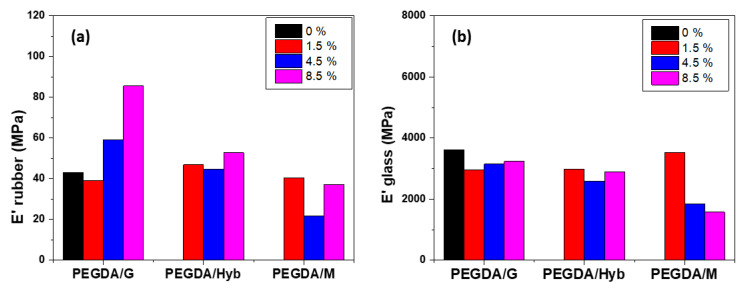
Storage modulus tensile at the rubbery (**a**) and the glassy state (**b**) obtained by DMA for neat and reinforced samples in different nanofiller’s percentage.

In DMA analysis (Figure 4), it was observed that in rubbery state, the storage modulus (E’) was improved by the addition of nanofillers, especially with graphene, in comparison with the neat PEGDA. Furthermore, E’ increased significantly when increasing the percentage of graphene in the composite. Contrary to this, samples with MWNTs showed a decrease in E’, whereas that of the hybrid showed an intermediate behavior and E’ increased slightly. Regarding the glassy state, all reinforced samples showed lower values of E’. Graphene-reinforced nanocomposites exhibited improved behavior in the thermomechanical tests compared with the neat polymer sample. However, samples reinforced with MWNTs showed a worse thermomechanical behavior, even in comparison with the neat PEGDA samples. The superiority of graphene is also presented in tensile tests, where E reaches an improvement up to 100% with 8.5%. Similar behavior was observed in TGA thermographs, whereby samples that contain graphene have a higher decomposition temperature.

The T_g_ of all the reinforced samples calculated by DMA tests showed a maximum decrease (~9 °C) compared to that of neat PEGDA, when the amount of nanofillers reaches 1.5% *w*/*w* (see Table 3). This behavior is caused by the weaker interactions between the polymer chains due to the intercalation of the nanofillers. In addition, in a previous work [23] it has been shown that the presence of the nanofillers has a negative effect in the light absorption and thus the polymerization degree, weakening the polymer network.

By increasing the percentage of the nanofillers at 4.5 or 8.5%, this difference between the T_g_ values of neat and reinforced samples was slightly lower (~5–9 °C) but still significant for PEGDA/G and PEGDA/Hyb samples. In the case of PEGDA/M, the T_g_ of the sample was comparable to that of the neat polymer. This change, when the percentage of the nanofiller increases, can be attributed to the contribution of the interactions between polymer chains and graphene surface which in general reduce the mobility of the polymer chains.

As concluded from all data, there are competitive phenomena that dominate each sample in different cases, depending on the tests that samples are subjected to and the percentage of the nanofiller. The first phenomenon is the absorption of UV radiation by the nanofillers which lowers the % DBC, leading to a less dense polymer network [23]. The second phenomenon is the decrease of the interactions between the polymer chains due to the intercalation of the nanofillers. The last phenomenon is the reduction of the mobility of the polymer chains, due to the van der Waals interactions between the polymer chains and the graphene surfaces that add to the thermomechanical properties.

## 3. Materials and Methods

PEGDA (average Mn = 575 g mol^−1^) was provided by Sigma Aldrich (St. Louis, MO, USA). The material 1-(4-(2-Hydroxyethoxy)-phenyl)-2-hydroxy-2-methyl-1-propane-1-one (Irgacure D2959) was used as a photo initiator and obtained by J&K Scientific (Beijing, China). MWNTs with an average aspect ratio of 100 and diameter between 30 and 50 nm were purchased by Sigma Aldrich and pristine graphene grade M with an average diameter of 25 μm by XG sciences (Lansing, MI, USA).

Samples preparation. PEGDA oligomer was mixed with photo initiator (PI) and stirred for 30 min at room temperature until a homogenous transparent liquid was achieved. Composite films were prepared according to the method described in our previous work [23].

Samples characterization. Scanning electron microscopy (SEM) was performed on a Zeiss EVO MA-10 electron microscope (Carl Zeiss AG, Oberkochen, Germany). DBC values were acquired by Fourier transform infrared spectroscopy (FT-IR, Shimadzu IR Tracer-100 spectrometer, Shimadzu, Kyoto, Japan) in the range of 500–4000 cm^−1^. The broad dielectric spectroscopy (BDS) technique requires samples that have a clear circle area with a minimum diameter of 22 mm. Thermogravimetric analysis (TGA) was carried out with a TA Instrument Q500 Thermo Gravimetric Analyzer under a nitrogen environment with a heating rate of 10 °C min^−1^ up to 500 °C. DMA was carried out with a Metravib DMA50. Samples used in dynamic mechanical analysis (DMA) tests were cut out of larger films with a sharp knife. The dimensions of DMA samples were 18 × 11 mm, and the thickness was dependent on sample type. Samples used in tensile tests had the same dimensions as DMA samples. Samples used for SEM, FTIR, DSC and TGA were obtained from the same sample that was used in BDS measurements.

## 4. Conclusions

In the present work, the thermomechanical behavior of carbon nanocomposite photopolymer films was studied. Graphene nanosheets, with high surface-to-thickness ratio, reinforce the nanocomposites, whereas MWNTs downgrade the samples in thermomechanical terms. Finally, nanocomposites with hybrid nanoparticles combine both electrical and thermomechanical advanced properties. More specifically, PEGDA/G showed the highest T_g_, and improved E’ in contrast with PEGDA/M samples. Upon tensile testing, performed in ambient temperature, all nanocomposites had improved E, compared with the neat polymer; however, graphene reinforced nanocomposites had the higher values over MWNTs.

Taking into account the conductivity behavior studied in the previous work [23], samples reinforced by hybrid nanoparticles appeared to have the best overall properties compared with the other nanocomposites and appeared to be more useful in feasible standalone conductive photopolymer films.

## Figures and Tables

**Table 1:** Samples of PEGDA reinforced with carbon nanostructures.

Carbon Nanofillers	PEGDA	PEGDA/G	PEGDA/M	PEGDA/Hyb
Mass percentage % *w*/*w*	0	1.5	1.5	1.5
	0	4.5	4.5	4.5
	0	8.5	8.5	8.5

**Table 2:** T_g_ (°C) of neat PEGDA, and composites PEGDA/G, PEGDA/M and PEGDA/Hyb at different percentage of nanofillers obtained by DSC.

Sample/Nanofiller	0%	1.5%	4.5%	8.5%
PEGDA/M	−22.5	−23.2	−31.1	−23.5
PEGDA/G		−19	−19.2	−14.6
PEGDA/Hyb		−21.3	−20	−21.2

**Table 3:** T_g_ (°C) of Neat PEGDA, and composites PEGDA/G, PEGDA/M and PEGDA/Hyb for different percentages of nanofillers obtained by DMA.

Sample/Nanofiller	0%	1.5%	4.5%	8.5%
PEGDA/M	−23.1	−32.6	−24.5	−24.3
PEGDA/G		−31.1	−27.5	−28
PEGDA/Hyb		−32.9	−30.2	−33.5

## Data Availability

Not applicable.

## Sample Availability

Samples of the compounds are not available from the authors.

## References

[1] Derby B. (2012). Printing and prototyping of tissues and scaffolds. Science.

[2] Dickens S.H., Stansbury J.W., Choi K.M., Floyd C.J.E. (2003). Photopolymerization Kinetics of Methacrylate Dental Resins. Macromolecules.

[3] Lalevee J., Fouassier J.P. (2015). Dyes and Chomophores in Polymer Science.

[4] Kumbaraci V., Aydogan B., Talinli N., Yagci Y. (2012). Naphthodioxinone-1,3-benzodioxole as photochemically masked one-component Type II photoinitiator for free radical polymerization. J. Polym. Sci. Part A.

[5] Brömme T., Schmitz C., Oprych D., Wenda A., Strehmel V., Grabolle M., Resch-Genger U., Ernst S., Reiner K., Keil D. (2006). Digital Imaging of Lithographic Materials by Radical Photopolymerization and Photonic Baking with NIR Diode Lasers. Chem. Eng. Technol..

[6] Sarantinos N., Loginos P., Charlaftis P., Argyropoulos A., Filinis A., Vrettos K., Adamos L., Kostopoulos V. (2019). Behavior of photopolymer fiber structures in microgravity. SN Appl. Sci..

[7] Allonas X., Pierrel J., Ibrahim A., Croutxé-Barghorn C. (2021). On-Demand Photopolymerization of Fiber-Reinforced Polymers Exhibiting the Shape Memory Effect. Polymers.

[8] Yun J.S., Park T.W., Jeong Y., Cho J.H. (2016). Development of ceramic-reinforced photopolymers for SLA 3D printing technology. Appl. Phys. A.

[9] Sahin M., Schlögl S., Kalinka G., Wang J., Kaynak B., Mühlbacher I., Ziegler W., Kern W., Grützmacher H. (2018). Tailoring the interfaces in glass fiber-reinforced photopolymer composites. Polymer.

[10] Liu Y., Lin Y., Jiao T., Lu G., Liu J. (2019). Photocurable modification of inorganic fillers and their application in photopolymers for 3D printing. Polym. Chem..

[11] Li Z., Chen H., Wang C., Chen L., Liu J., Liu R. (2018). Efficient photopolymerization of thick pigmented systems using upconversion nanoparticles-assisted photochemistry. J. Polym. Sci. Part A Polym. Chem..

[12] Sangermano M., Marchi S., Valentini L., Bon S.B., Fabbri P. (2011). Transparent and Conductive Graphene Oxide/Poly(ethylene glycol) diacrylate Coatings Obtained by Photopolymerization. Macromol. Mater. Eng..

[13] Kasraie M., Abadi P.P.S.S. (2021). Additive manufacturing of conductive and high-strength epoxy-nanoclay-carbon nanotube composites. Addit. Manuf..

[14] Xu Y., Li Y., Hua W., Zhang A., Bao J. (2016). Light-weight silver plating foam and carbon nanotube hybridized epoxy composite foams with exceptional conductivity and electromagnetic shielding property. ACS Appl. Mater. Interfaces.

[15] Wu Q., Zou S., Gosselin F.P., Therriault D., Heuzey M.C. (2018). 3D printing of a selfhealing nanocomposite for stretchable sensors. J. Mater. Chem. C.

[16] Scordo G., Bertana V., Scaltrito L., Ferrero S., Cocuzza M., Marasso S.L., Romano S., Sesana R., Catania F., Pirri C.F. (2019). A novel highly electrically conductive composite resin for stereolithography. Mater. Today Commun..

[17] Ryan K.R., Down M.P., Hurst N.J., Keefe E.M., Banks C.E. (2022). Additive manufacturing (3D printing) of electrically conductive polymers and polymer nanocomposites and their applications. eScience.

[18] Kwok S.W., Goh K.H.H., Tan Z.D., Tan S.T.M., Tjiu W.W., Soh J.Y., Glenn Ng Z.J., Chan Y.Z., Hui H.K., Goh K.E.J. (2017). Electrically conductive filament for 3D-printed circuits and sensors. Appl. Mater. Today.

[19] Kostagiannakopoulou C., Loutas T.H., Sotiriadis G., Markou A., Kostopoulos V. (2015). On the interlaminar fracture toughness of carbon fiber composites enhanced with graphene nano-species. Compos. Sci. Technol..

[20] Zhang H., Bilotti E., Tu W., Lew C.Y., Peijs T. (2015). Static and dynamic percolation of phenoxy/carbon nanotube nanocomposites. Eur. Polym. J..

[21] Kernin A., Wan K., Liu Y., Shi X., Kong J., Bilotti E., Peijs T., Zhang H. (2019). The effect of graphene network formation on the electrical, mechanical, and multifunctional properties of graphene/epoxy nanocomposites. Compos. Sci. Technol..

[22] Feng Z., Li Y., Xin C., Tang D., Xiong W., Zhang H. (2019). Fabrication of Graphene-Reinforced Nanocomposites with Improved Fracture Toughness in Net Shape for Complex 3D Structures via Digital Light Processing. C J. Carbon Res..

[23] Loginos P., Patsidis A., Georgakilas V. (2020). UV-Cured Poly(Ethylene Glycol) Diacrylate/Carbon Nanostructure Thin Films. Preparation, Characterization, and Electrical Properties. J. Compos. Sci..

[24] Nam K.H., Cho J., Yeo H. (2018). Thermomechanical Behavior of Polymer Composites Based on Edge-Selectively Functionalized Graphene Nanosheets. Polymers.

[25] Hajian M., Reisi M.R., Koohmareh G.A., Jam A.R.Z. (2012). Preparation and characterization of Polyvinylbutyral/Graphene Nanocomposite. J. Polym. Res..

